# Role for Krüppel-Like Transcription Factor 11 in Mesenchymal Cell Function and Fibrosis

**DOI:** 10.1371/journal.pone.0075311

**Published:** 2013-09-17

**Authors:** Angela Mathison, Adrienne Grzenda, Gwen Lomberk, Gabriel Velez, Navtej Buttar, Pamela Tietz, Helen Hendrickson, Ann Liebl, Yuning Y. Xiong, Gregory Gores, Martin Fernandez-Zapico, Nicholas F. LaRusso, William Faubion, Vijay H. Shah, Raul Urrutia

**Affiliations:** 1 Laboratory of Epigenetics and Chromatin Dynamics, Mayo Clinic, Rochester, Minnesota, United States of America; 2 Division of Gastroenterology and Hepatology, Mayo Clinic, Rochester, Minnesota, United States of America; 3 Division of Oncology Research, the Schulze Center for Novel Therapeutics, Mayo Clinic, Rochester, Minnesota, United States of America; 4 Translational Epigenomics Program, Center for Individualized Medicine, Mayo Clinic, Rochester, Minnesota, United States of America; 5 Departments of Biophysics, Medicine, Biochemistry and Molecular Biology, Mayo Clinic, Rochester, Minnesota, United States of America; University of Bergen, Norway

## Abstract

Krüppel-like factor 11 (KLF11) and the highly homologous KLF10 proteins are transcription factors originating from duplication of the *Drosophila melanogaster* ancestor *cabut*. The function of these proteins in epithelial cells has been previously characterized. In the current study, we report a functional role for KLF11 in mesenchymal cells and in mesenchymal cell dysfunction, namely, fibrosis, and subsequently perform a detailed cellular, molecular, and *in vivo* characterization of this phenomenon. We find that, in cultured mesenchymal cells, enhanced expression of KLF11 results in activated extracellular matrix pathways, including collagen gene silencing and matrix metalloproteinases activation without changes in tissue inhibitors of metalloproteinases. Combined, reporter and chromatin immunoprecipitation assays demonstrate that KLF11 interacts directly with the collagen 1a2 (*COL1A2*) promoter in mesenchymal cells to repress its activity. Mechanistically, KLF11 regulates collagen gene expression through the heterochromatin protein 1 gene-silencing pathway as mutants defective for coupling to this epigenetic modifier lose the ability to repress *COL1A2*. Expression studies reveal decreased levels of KLF11 during liver fibrogenesis after chemically induced injury *in vivo*. Congruently, KLF11^-/-^ mice, which should be deficient in the hypothesized anti-fibrogenic brake imposed by this transcription factor, display an enhanced response to liver injury with increased collagen fibril deposition. Thus, KLFs expands the repertoire of transcription factors involved in the regulation of extracellular matrix proteins in mesenchymal cells and define a novel pathway that modulates the fibrogenic response during liver injury.

## Introduction

Studies on KLF10 and KLF11, members of the same subfamily of KLF transcription factors, have extended the understanding of how GC-rich sites in proximal promoters are regulated. The two proteins regulate gene expression programs in numerous biological processes conserved from *Drosophila melanogaster* to humans. Moreover, disruptive alterations in these proteins and their regulation are found in a variety of human diseases [1,2,3,4,5]. Thus, the careful biochemical characterization of these proteins possesses biological and biomedical relevance.

Structurally, KLF10 and KLF11 are characterized by three highly conserved C_2_H_2_ zinc fingers at the C-terminus, which recognize and bind GC-rich sequences in gene promoters. Variable N-terminal domains recruit chromatin-remodeling co-regulators that dictate their function as activators, repressors, or both. In fact, these proteins can regulate the expression of a multitude of gene networks by differentially coupling to histone acetyltransferases (HATs), deacetyletransferases (HDACs), and methyltransferases (HMTs). Protein depletion or dysfunction, as such, may result in widespread effects.

Biochemical studies using both cell and animal models reveal that KLF10 and 11 proteins are involved in regulating the growth and differentiation of epithelial cells with disruption associated to neoplastic development. KLF10 is critical for the physiological homeostasis of mesenchymal cells, including the cellular components of tendons, bone, and muscle. A role for KLF11 in mesoderm-derived cells and pathological processes, however, has remained elusive.

Liver fibrosis is an ideal model system for investigating the pathobiological effects of altered KLF11 levels on cells of mesenchymal origin. The fibrogenic process is initiated with the clearance of degraded hepatocytes and finalized by the secretion of collagen fibrils by hepatic stellate cells (HSCs) [6]. In the setting of chronic injury, the fibrotic cycle is initiated and interrupted multiple times, leading to excess fibril deposition that progressively impairs hepatic function [7]. Given the known functional repertoire of KLF10 and KLF11, these proteins likely mediate numerous aspects of the fibrogenic response.

Thus, an in-depth investigation into how KLF11 affects mesenchymal cells is critical to fill a gap in the existing knowledge of the biological and potential pathophysiological functions of these transcriptional regulators. Our *in vitro* and *in vivo* data indicates that, in liver mesenchymal cells, KLF11 regulates the expression of extracellular matrix genes and that its absence yields a more robust fibrogenic response to injury. This is the first evidence that KLF11 modulates fibrogenic responses in mesenchymal cells. As mutations in KLF11 pathways have been found to cause human disease, this information should be considered in other pathophysiological processes associated to this gene.

## Materials and Methods

### Ethics statement

All animal experiments were performed per the recommendations outlined in the Guide for Care and Use of Laboratory Animals from the National Institutes of Health as required by Mayo Clinic. These guidelines were incorporated into the current study protocol (#A34612), which was reviewed and approved by the Institutional Animal Care and Use Committee (IACUC), at Mayo Clinic, Rochester, MN.

### Cell culture

Human hepatic stellate cell line (LX2) was a generous gift from Dr. Scott Friedman (Mount Sinai, NY) [8]. Cells were cultured as previously described [9].

### Plasmids and recombinant adenovirus

Studies used full-length KLF11, KLF11-EAPP, and KLF11ΔHP1 pcDNA3.1/His (Invitrogen) constructs and epitope-tagged (6XHis-XpressTM) KLF11, KLF11ΔHP1, and empty vector (Ad5CMV) adenovirus as previously described [10,11].

### Antibodies

Primary antibodies were purchased from the following companies: polyclonal α-smooth muscle actin (αSMA) from Abcam (Cambridge, MA), polyclonal collagen I from Abcam, monoclonal OMNI-D8 from Santa Cruz (Santa Cruz, CA), and monoclonal (clone B-5-1-2) α-tubulin from Sigma.

### Western blot

LX2 cells (2x10^6^) were plated and infected as described above. After 48 hours, cells were lysed in RIPA buffer (1X lysis buffer-150 with protease inhibitors) for collagen I measurement or lammeli buffer for KLF11 and α-tubulin measurements. Proteins were separated by 10% SDS/PAGE, transferred to membrane, blocked with 5% milk and probed overnight with primary antibodies at the following dilutions: collagen I (1:5000), OMNI-D8/His (1:1000), and α-tubulin (1:1000). Blots were washed and incubated with HRP-conjugated secondary antibodies (1:5000) and developed with chemiluminescence.

### RT-PCR and transcriptional profiling of stellate cells

LX2 cells (2x10^6^) were plated and infected with empty vector, KLF11, and KLF11ΔHP1 adenovirus (multiplicity of infection, 250:1). After 48 hours, RNA was isolated (biological triplicate) as described and a 233 gene expression panel compared utilizing SABioscience’s Extracellular Matrix and Adhesion (PAHS-013), TGF/BMP (PAHS-035), and Growth Factor (PAHS-041) quantitative PCR Arrays.

### Chromatin Immunoprecipitation (ChIP)

LX2 cells (2x10^6^) were plated and infected with empty vector and KLF11 adenovirus (multiplicity of infection, 250:1). Immunoprecipitation was completed as previously described using the Magna ChIP G kit (Upstate) and an antibody against the His-tagged KLF11 protein (OMNI-D8) [10,11]. Briefly, protein-DNA complexes were collected using magnetic protein G beads, wash buffers eliminated non-specific binding, and complexes were eluted and reverse crosslinked with ChIP elution buffer and proteinase K. DNA samples were purified and primers designed against a region of the collagen 1A2 promoter upstream of the transcriptional start site were used to consider the promoter occupancy by KLF11. Primers were synthesized by Integrated DNA Technologies (Coralville, IA): 5'-CCTTTCAAACCTAGGGCCTGG-3' (forward) and 5'-TCCTAGCTTGCCTTTGCTGAGG-3' (reverse). PCR on col1a2 target promoter was performed using Platinum Taq (Invitrogen) according to manufacturer’s protocol with the 33 cycles of: 94C for 15s, 55C for 30s, 72C for 2min. Products were examined on a 1.5% agarose gel [10,11].

### Reporter assays

Collagen 1a2 promoter activity [12] was monitored via luciferase reporter assay. LX2 cells (2x10^6^) were resuspended in 250 µL growth media, mixed with 3 µg of promoter construct and 9µg of each empty vector, KLF11, KLF11-EAPP, and KLF11-ΔHP1 and electroporated on α BTX Model 820 square wave electroporator at 310V, 1 pulse for 10ms. Protein was isolated 24 hours post-electroporation and relative luciferase expression was assayed using Dual Luciferase Reporter Assay System (Promega) on a Berthold Lumat LB 9507 luminometer. Luciferase activity was controlled by total protein concentration. Experiments were performed in duplicate.

### CCl_4_ Administration and Sample Collection

Mayo Clinic Institutional Animal Care and Use Committee (#A34612) approved all procedures. KLF11^-/-^ mice were generated by standard homologous recombination techniques and backcrossed in our laboratory to C57Bl/6 for greater than 20 generations [13]. Wild type and KLF11^-/-^ mice were treated by intraperitoneal injection of carbon tetrachloride (CCl_4,_ Sigma-Aldrich, St. Louis, MO) to induce liver fibrosis as previously described [14,15]. Control animals received olive oil (OO). Animals were sacrificed at 6-7 weeks. A total of 14 wild type and 17 KLF11^-/-^ were treated with CCl_4_, while 8 wild type and 7 KLF11^-/-^ mice were treated with OO. Treatment groups were gender balanced.

### RT-PCR and transcriptional profiling of liver tissues

Total RNA was extracted from liver tissue using an RNeasy kit (Qiagen). For qPCR arrays, cDNA was synthesized from 2µg of pooled RNA using the RT2 First Strand kit (Qiagen). Transcript levels were compared between conditions utilizing SABioscience’s pathway focused Fibrosis Arrays (PAMM-120). For analysis of individual fibrosis genes, RNA (1µg) was converted to cDNA using an oligo (dT) primer and SuperScript III First-Strand Synthesis System for RT-PCR (Invitrogen) as per manufacturer’s protocol. Real time PCR was performed using RT² SYBR® Green qPCR Mastermixes (Qiagen) and RT² qPCR Primers (Qiagen) designed against α2 smooth muscle actin (ACTA2), collagen 1a1 (COL1A1), collagen 1a2 (COL1A2), Krüppel-like factor 11 (KLF11) and controlled with housekeeping genes beta-actin (ACTB) and glyceraldehyde-3-phosphate dehydrogenase (GAPDH). PCR cycles were completed with the Bio-Rad CFX96 real-time PCR machine. Fold changes were calculated using Bio-Rad CFX manager or SABioscience’s RT2 Profiler PCR Array Data Analysis software.

### Immunohistochemistry and TUNEL assay

Formalin-fixed liver tissues were paraffin-embedded, sectioned (5 µm), and stained with hematoxylin and eosin (H&E), Sirius red, or Masson trichrome by the Mayo Clinic Histology Core (Scottsdale, AZ). Electron microscopy samples were prepared and imaged by the Mayo Electron Microscopy Core at 80kV. For immunohistochemistry, rehydrated sections were unmasked by incubation in sodium citrate buffer at 95°C for 20 minutes. Sections were quenched for endogenous peroxidase with 3% hydrogen peroxide, avidin/biotin blocked (Vector laboratories), and pre-treated with CAS Block (Invitrogen, Grand Island, NY) prior to overnight incubation with primary antibodies at 4°C with collagen I or αSMA (1:100). Sections were then incubated with biotinylated goat anti-rabbit (Vector, 1:200) and HRP-streptavidin (Invitrogen). Immunoreactivity was monitored by Nova Red development (Vector) and slides counterstained with hematoxylin (Sigma). TUNEL analysis was carried out using the ApopTag Peroxidase in situ cell apoptosis detection kit (Millipore) according to the manufacturer’s directions. The color reaction was developed with Nova Red (Vector) and the sections were counterstained with hematoxylin, as above, prior to analysis by light microscopy. Images for both immunohistochemistry and TUNEL assays were captured with an AxioPlan2 upright microscope (Zeiss, Thornwood, NY) at a magnification of X200 then quantified by the KS400 3.0 Imaging System (Zeiss). 10 random field views centered on the central vein per animal per treatment were selected in a blinded fashion as to their affiliation to an experimental group and averaged to yield a final relative value (n ≥ 5 averaged relative values pre animal and treatment).

### Serum analysis

Whole blood was isolated from CCl_4_ and OO treated KLF11^-/-^ and wild type mice (n=3 per condition), treated with lithium heparin and plasma isolated after centrifugation for 3 minutes at room temperature. Serum was mixed 1:1 with saline and tested with profile cartridges on the Abaxis VetScan VS2, specifically the Mammalian Liver Profile and Comprehensive Diagnostic Profile. Resultant values were multiplied by two to recapitulate values present in the whole plasma.

### Statistical analysis

Significance level was set at p<0.05 for all experiments and determined by one-way analysis of variance (ANOVA) and Tukey post-hoc multiple comparison tests using GraphPad Prism 6.

## Results

### Studies in cultured liver mesenchymal cells reveal a role for KLF11 in the regulation of extracellular matrix remodeling

**Figure 1:**
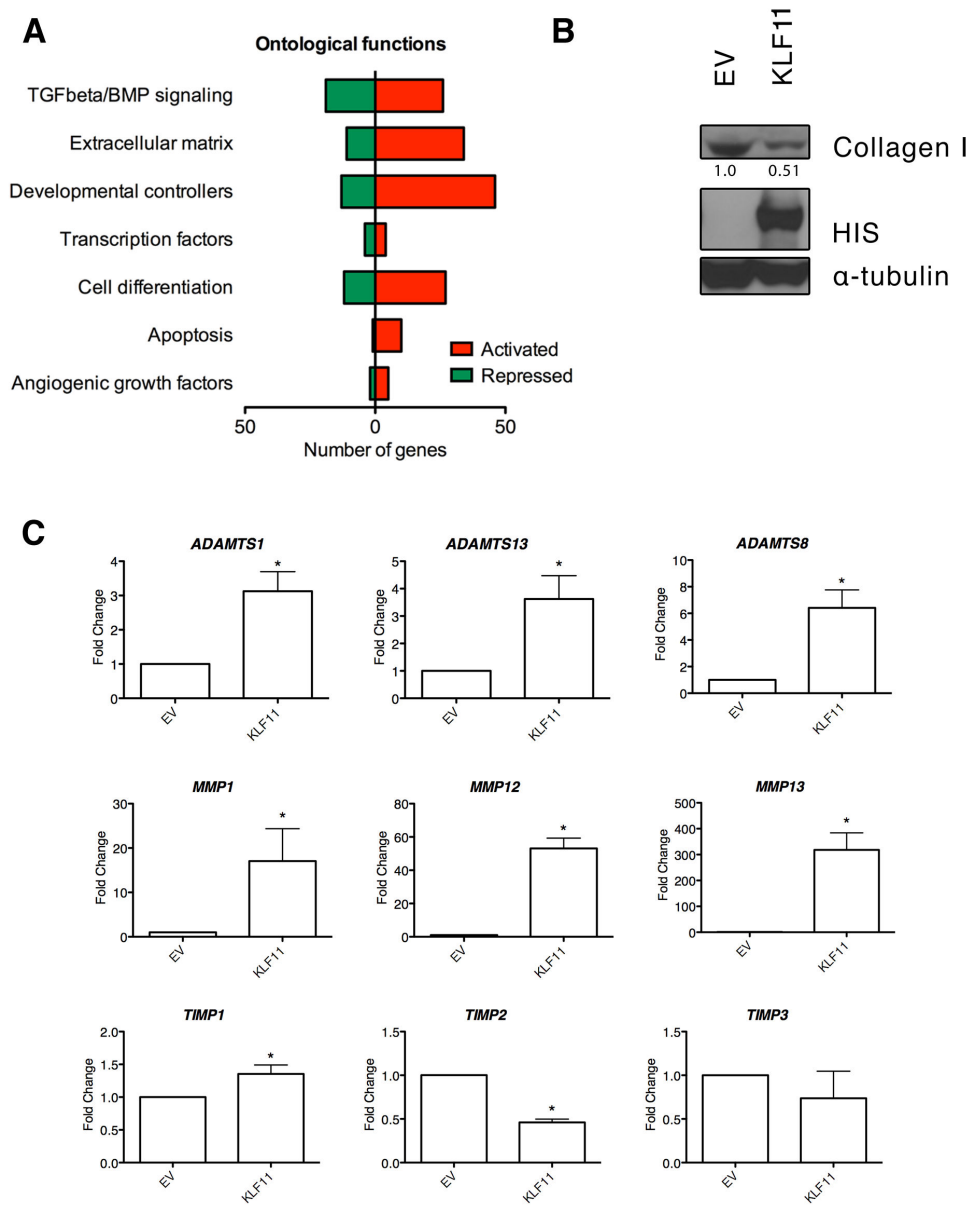
KLF11 is critical to a variety of biological processes in liver mesenchymal cells. (A) LX2 cells overexpressing KLF11 elicit a variety of changes in extracellular matrix, TGFβ response, and growth factor genes. The biosynthetic response of 232 unique genes was measured by qPCR arrays (SA Biosciences) in LX2 hepatic stellate cells comparing empty vector (EV) and KLF11 transduced cells after 48 hours. The full list of altered genes may be found in Table S1. The number of genes significantly regulated by KLF11 compared to empty vector control across a variety of ontological categories is represented here. (B) Protein levels of collagen are decreased in response to KLF11 overexpression in LX2 cells. Collagen I levels were measured by western blot in LX2 hepatic stellate cells comparing EV and KLF11 transduced cells after 48 hours of infection. In the same samples, overexpression of His-KLF11 was confirmed by Omni-D8 western blot and α-tubulin confirms equal loading of lysates. Relative densitometry of collagen I levels is shown, normalized to α-tubulin control for each sample. (C) Transcript levels of matrix metalloproteinases are greatly induced while their regulators, TIMPs, are generally unchanged or downregulated. For KLF11 overexpression compared to empty vector: *ADAMTS1*, 3.12 ± 0.57; *ADAMST13*, 3.62 ± 0.85; *ADAMTS8*, 6.41 ±1.35; *MMP1*, 17.07 ± 7.30; *MMP12*, 53.08 ± 6.24; *MMP13*, 318.10 ± 65.98; *TIMP1*, 1.35 ± 0.14; *TIMP2*, 0.46 ± 0.04; *TIMP3*, 0.74 ± 0.31. * p-value <0.05.

To gain insight into the gene program regulated by KLF11 in mesenchymal cells, we analyzed adenoviral-based overexpression of this transcription factor in a cultured hepatic stellate cell line in conjunction with a 233 gene expression panel spanning growth factors, signaling pathways, adhesion molecules, extracellular matrix regulators, apoptosis molecules, and angiogenic mediators, among others (Figure 1A, Table S1). Of the genes studied, 40% (93 genes) were increased greater than 2 fold following KLF11 overexpression in contrast to only 13% with decreased (-1.5 fold or less) expression.

In this broad panel of biological functions, we noted a particular enrichment in extracellular matrix and cellular adhesion proteins (Figure 1A). In particular, the major gene encoding fibrillar collagen type I in extracellular matrix, Collagen 1a2, was significantly downregulated, a finding subsequently confirmed by Western blot (Figure 1B). Furthermore, we found a variety of metalloproteinases (MMPs) with increases ranging from 3 fold for ADAMTS1 to 318 fold for MMP13 (Figure 1C). Notably, we also observed that tissue inhibitors of matrix metalloproteinases (TIMPs) were generally unchanged or downregulated (Figure 1C). These data reveal that high levels of KLF11 trigger a molecular response that leads to suppression of extracellular matrix remodeling and collagen deposition.

**Figure 2:**
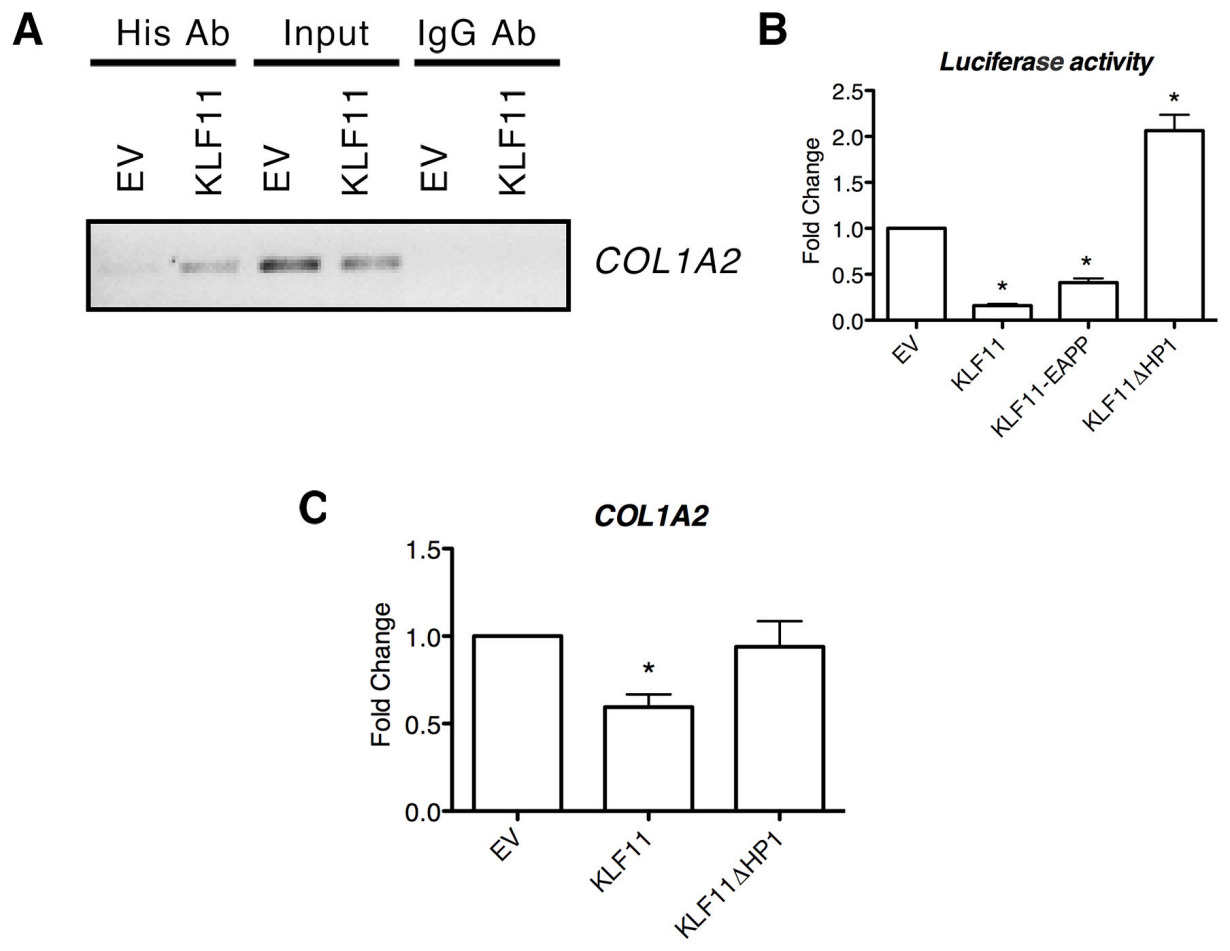
Collagen 1 is directly repressed by KLF11 in mesenchymal cells coupled to the HP1-HMT pathway. (A) To illustrate that KLF11 was a direct regulator of the collagen I promoter, chromatin immunoprecipitation was performed on LX2 cells transduced with empty vector or KLF11. Immunoprecipitation of KLF11 displayed amplification of the collagen 1a2 promoter by PCR, thus verifying the direct binding of KLF11 to the promoter of collagen1a2. Input controls for empty vector and KLF11 are included to ensure equal volume of precipitated DNA as well as the results of amplification from immunoprecipitates using a non-specific IgG antibody. (B) Further evidence that collagen is a direct target of the sequence specific transcription factor KLF11 was observed in luciferase assays. Sections of the collagen 1a2 promoter containing GC consensus sequences were cloned upstream of a luciferase reporter. The reporter gene was significantly repressed upon overexpression of KLF11 in LX2 cells (84 ± 1.94% compared to empty vector, p<0.05). Examination of the effect of overexpression of KLF11-EAPP mutant reveals only a slight change in the repression of the collagen I promoter (41 ± 4.5% repression compared to empty vector, p<0.05). However, overexpression of the KLF11ΔHP1 mutant lead to completion de-repression of the collagen I promoter (206 ± 17% compared to empty vector, p<0.05), indicating that the repression of the gene occurs through the HP1-HMT chromatin remodeling pathway. (C) PCR of collagen 1a2 expression in LX2 cells transduced with empty vector, KLF11, or KLF11ΔHP1 demonstrate that expression of the gene is repressed in the presence of KLF11 (0.59 ± 0.11 fold compared to empty vector) but depressed in the presence of the inactivating KLF11ΔHP1 mutant (0.92 ± 0.14 fold compared to empty vector).

Although the gamut of effects mediated by KLF11 may require the full interaction of all of the genes found differentially expressed by our transcriptional analyses, the detailed characterization of the entire gene network is impractical. As collagen fibril secretion is one of the primary functions of hepatic stellate cells, we selected the Collagen 1 gene for more detailed biochemical analysis to determine if the effects of KLF11 on its transcriptional regulation are directly or indirectly modulated. To test if KLF11 binds the promoter of the *COL1A2* gene, chromatin immunoprecipitation (ChIP) was performed. KLF11 binds to the endogenous *COL1A2* promoter compared to empty vector control, which demonstrates no binding (Figure 2A). Additionally, luciferase reporter assays was performed and demonstrated that KLF11 downregulates *COL1A2* promoter activity by 84 ± 1.94% compared to empty vector (Figure 2B). Collectively, these data reveal that one of the functions of KLF11 in mesenchymal cells is the direct regulation of extracellular matrix remodeling by coupling to the promoters of genes, functioning as both an activator and a repressor of gene expression.

### KLF11 directly downregulates collagen I gene expression by coupling to distinct chromatin remodeling pathways

KLF11 functions in transcriptional regulation as a scaffold in the recruitment of various chromatin pathways. Certain pathways, such as the Sin3a and HP1 pathways, can both mediate the silencing responses of KLF11 while others, like HATs, mediate activation in different contexts [10,11]. Thus, to gain insight into the chromatin-mediated mechanisms used by KLF11 to regulate the expression of collagen genes, we used loss of function mutants. One these mutants, KLF11-EA29,30PP, disrupts the interaction between KLF11 and Sin3a, disrupting HDAC recruitment [10,11]. A second mutant, KLF11ΔHP1, abrogates the repressive interaction between KLF11 and all isoforms of HP1, thereby preventing the recruitment of the G9a and SUV39 HMTs [10]. Interestingly, collagen repression was not significantly altered in the presence of the KLF11-EA29,30PP mutant, demonstrating that the Sin3/HDAC pathway likely does not participate in this process (Figure 2B). However, repression was completely reversed in the presence of the KLF11ΔHP1 mutant, indicating that gene silencing of *COL1A2* occurs through the HP1-HMT pathway [10,11]. Expression analysis of *COL1A2* under conditions of KLF11 and KLF11ΔHP1 overexpression confirmed this observation (Figure 2C). Together, these experiments indicate that one important mechanism by which KLF11 performs some of its important extracellular remodeling functions is through the coupling of KLF11 to the HP1-HMT pathway.

### KLF11 is downregulated *in vivo* after chemically-induced liver injury

**Figure 3:**
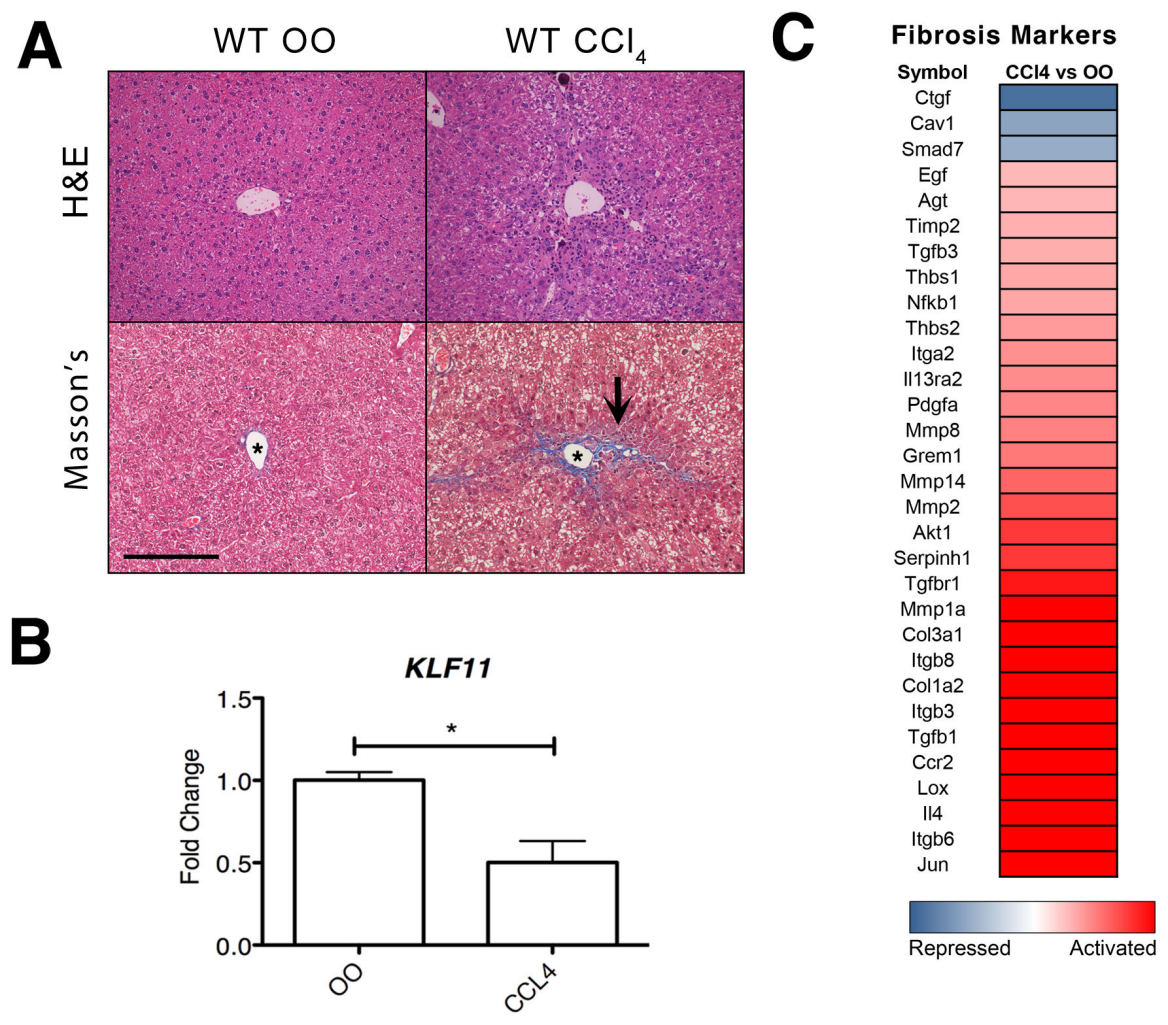
KLF11 is repressed during liver injury caused by chronic CCl_4_ exposure. (A) C57Bl/6 wild type mice were treated with CCl_4_ or olive oil (OO) for a period of six weeks. Liver sections with hematoxylin and eosin (H and E) labeling are provided to demonstrate overall tissue architecture. In CCl_4_-treated mice, Masson’s trichrome staining indicates increase extracellular matrix accumulation (bright blue) around the central vein. All images are X200 magnification with scale bar equaling 200µm. Asterisk in image denotes central vein. Black arrows denote C-C septa. (B) In wild type mice, levels of KLF11 expression were measured by qPCR. Data are expressed as fold change over olive oil control after normalization to housekeeping genes beta-actin (ACTB) and glyceraldehyde-3-phosphate dehydrogenase (GAPDH). The data are means ± SEM, n=4 to 6 per experimental condition. Mice treated with CCl_4_ had significantly downregulated (0.5 ± 0.13) levels of KLF11 compared to OO-treated control animals (* = p<0.05). (C) qPCR measured the changes in 31 fibrosis markers in pooled liver samples from wild type mice treated with CCl_4_ for six weeks normalized to OO control. All genes with a fold change <-2 and >2.0 are considered differentially regulated between the CCl4 and OO treatments. Scaling is from -5 fold change (repressed) to 1 (unchanged) to +5 fold change (activated).

Our *in vitro* data indicates that KLF11 gene regulation is critical to the normal physiological function of mesenchymal cells, in particular, in processes involved in extracellular matrix remodeling. To determine if deregulation of KLF11 protein leads to pathological effects in the functions of these cells, we sought to determine if liver injury modulates KLF11 expression levels. Fibrosis induction in the liver through exposure to carbon tetrachloride (CCl_4_) is a highly reproducible experimental model that affects all liver cell types and characterized by an activation of mesenchymal cells in response to the chemical stimuli [16]. As control of the treatment, we determined the extent of fibrosis elicited by CCl_4_ via staining liver sections with Masson’s trichome staining (bright blue), which permits the visualization of ECM deposition (e.g. collagens) against the background of the entire tissue (keratin and muscle fibers in red, cytoplasm in pink). Figure 3A indicates an increase in extracellular material in wild type CCl_4_-treated animals compared to OO-treated controls. As previously described [15,17], the area around the central vein in the CCl_4_-treated animals displayed the greatest amount of ECM deposition and thin collagen fiber tracts were observed to extend between central veins (C-C septa), forming the edge of the developing pseudo-nodule as well as between the central vein and the portal tract (C-P septa). RNA from whole livers of these animals was used to evaluate the expression of KLF11 and markers of a fibrogenic response. As shown in Figure 3B, KLF11 expression was significantly decreased (0.5 ± 0.13, p<0.05) as compared to OO treated. The decrease of KLF11 mRNA was accompanied by significant changes in the expression of 31 fibrotic markers in CCl_4_-treated animals as compared to OO treated controls (Figure 3C). Taken together, this data demonstrates that a downregulation in the level of KLF11 mRNA is associated with the cellular and molecular responses contributing to the genesis of liver fibrosis.

### Inactivation of KLF11 enhances the severity of liver fibrosis in response to chemically-induced injury

**Figure 4:**
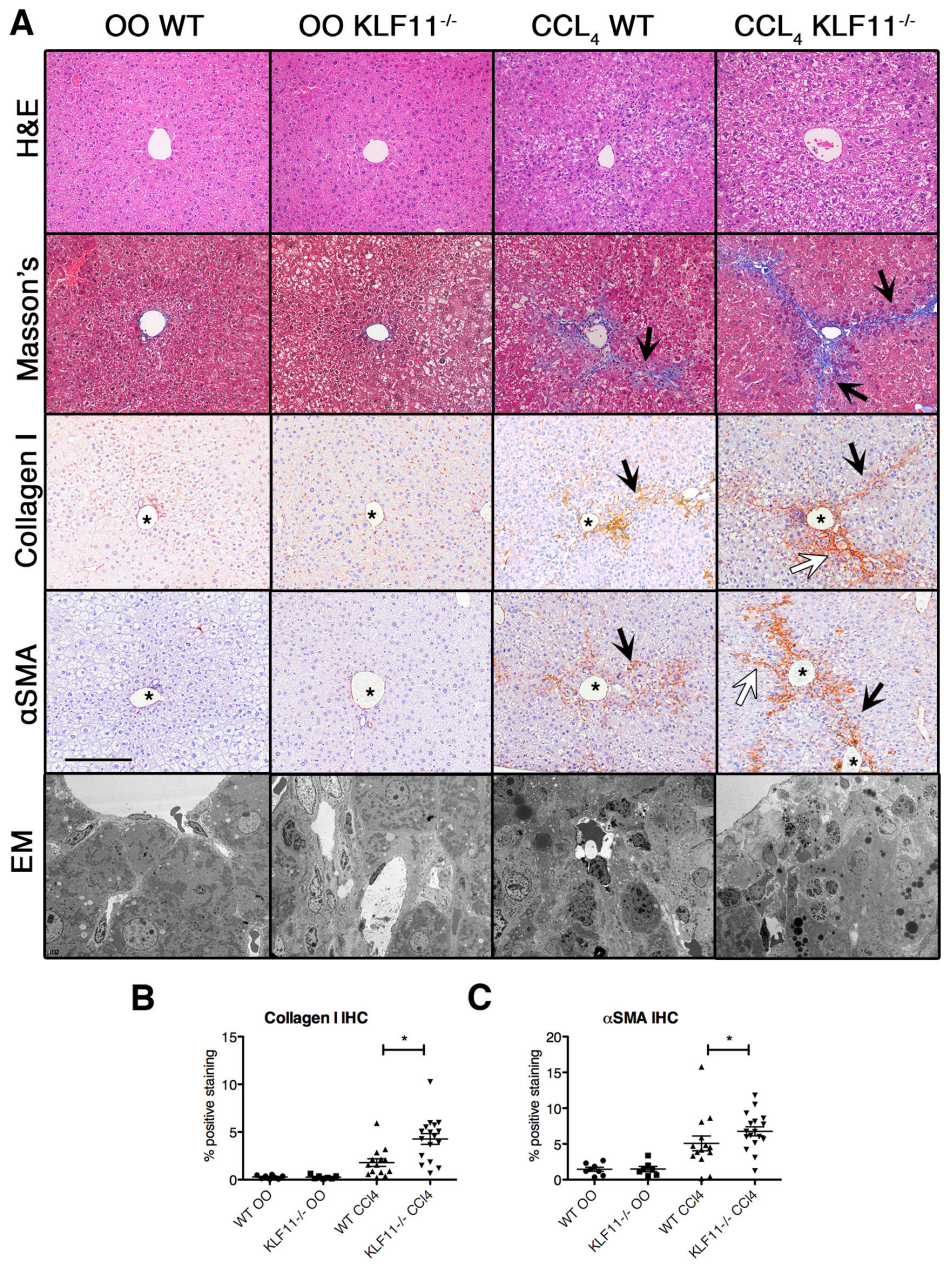
Inactivation of KLF11 correlates with an increased severity of fibrosis. (A) C57Bl/6 wild type mice were treated with CCl_4_ or olive oil (OO) control for a period of six weeks. The extent of fibrosis was observed with Masson’s trichrome staining with no fibrosis observed under olive oil treatment in either KLF11^-/-^ or wild type mice. In contrast, fibrosis was observed in CCl_4_-treated wild type mice as indicated by an increase in extracellular matrix deposition. Collagen I immunohistochemistry reveals similar increases in fibrous septa formation. Similarly, the stellate cell marker α-smooth muscle actin (αSMA) appears increased in CCl_4_-treated wild type mice compared to OO-treated wild type mice. CCl_4_-treated KLF11^-/-^ mice display significant increase in αSMA and collagen I labeling as compared to CCl_4_-treated wild type. There is an increase in the extent of C-C septa formation as well as the presence of C-P septa in CCl_4_-treated KLF11^-/-^ mice compared to wild type, indicating a more severe fibrotic response to the chemical insult. Electronic microscopy also revealed disrupted cellular architecture in the CCl_4_-treated KLF11^-/-^ and wild type mice compared to OO-treated controls. All images are X200 magnification with scale bar equaling 200µm. Black arrows indicated C-C septa and white arrows denote C-P septa. Asterisks label the central vein. (B-D) Masson’s trichrome, collagen I, and α-smooth muscle actin (αSMA) staining were quantified using *Imaging* *System* *KS400* as detailed in the materials and methods. The levels of all markers are significantly increased in the CCl_4_-treated treated animals and the CCl_4_-treated KLF11^-/-^ mice have significantly high percentage of αSMA and collagen I staining than the similarly treated wild type animals (* = p<0.05). Each datapoint represents the average of the intensity values of 10 random fieldviews.

The phenotype of our KLF11^-/-^ mouse model has yet to be fully characterized, beyond a deficiency in pancreatic cell secretion [13]. Histopathological characterization of livers from these animals shows no significant difference with the wild-type C57Bl/6 animals (Figure 4A). To test our hypothesis that KLF11 serves as a gene modifier of the HSC response to chronic CCl_4_ exposure, we used our genetically engineered mouse model. Thus, wild type C57Bl/6 and KLF11^-/-^ littermates were treated with CCl_4_ or OO as before and the livers harvested at six weeks. As shown in Figure 4A, Mason’s trichrome staining demonstrated an increase in central vein fibrosis and C-C septa formation in wild type mice following chronic CCl_4_ treatment compared to OO treated controls. However, the extent of C-C septa formation was notably increased in the CCl_4_-treated KLF11^-/-^ mice, which possessed highly established pseudo-lobule borders and evidence of C-P septa formation, indicating a more severe fibrotic response to the chemical insult. To more precisely access the difference in collagen deposition, immunohistochemistry using an antibody against collagen I (Figure 4A) recapitulated the observations from the Mason’s trichrome labeling. Digital quantification of the collagen staining revealed that the percentage of collagen I deposition in CCl_4_-treated KLF11-/- mice was significantly increased (4.27% mean, p<0.05) compared to CCl_4_-treated wild type mice treated with CCl_4_ (1.47% mean, Figure 4B). Thus, this observation in the KLF11^-/-^ mouse further supports the role of KLF11 in collagen gene regulation.

### Inactivation of KLF11 enhances mesenchymal cell activity in the setting of fibrosis

**Figure 5:**
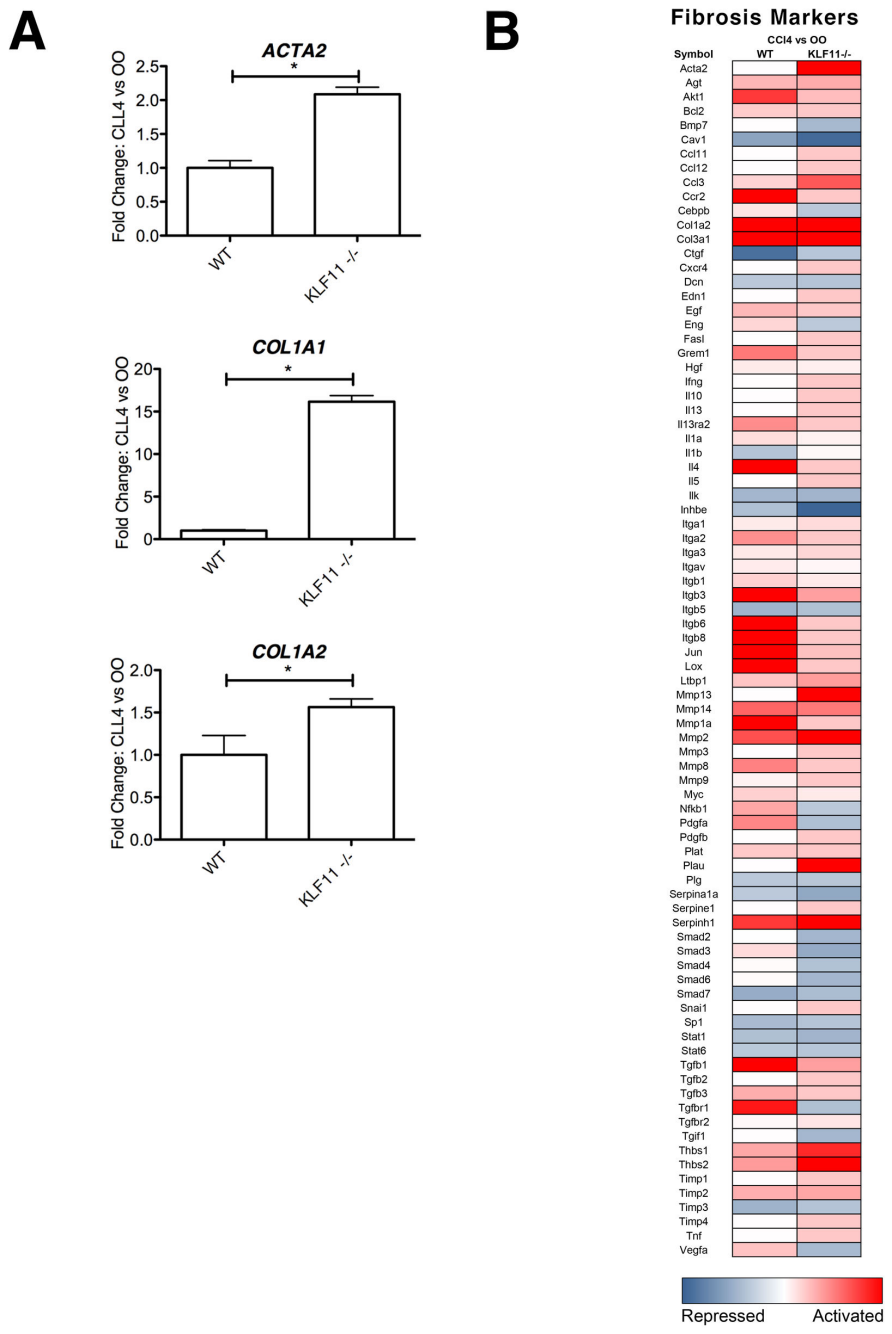
Mesenchymal cell activation and fibril deposition are increased in response to chronic CCl_4_ treatment in the absence of KLF11. (A) In a subset of CCl_4_-treated KLF11^-/-^ mice and CCl_4_-treated wild type mice, *ACTA2*, *COL1A1*, and *COL1A2* transcript levels were measured by PCR, validating the values obtained by the fibrotic marker array depicted in part B and the data for which appear in Table S2. Each of these genes was found to be upregulated in CCl_4_-treated KLF11^-/-^ mice as compared to wild type control. Data are expressed as fold change over olive oil control for each genotype after normalization to housekeeping genes beta-actin (ACTB) and glyceraldehyde-3-phosphate dehydrogenase (GAPDH). *ACTA2* (KLF11^-/-^, 2.09 ± 0.10 compared to WT). *COL1A1* (KLF11^-/-^: 16.16 ± 0.72 compared to WT). *COL1A2* (KLF11^-/-^: 1.56 ± 0.10 compared to WT). * = p<0.05. (B) qPCR array measured the changes in 84 fibrosis makers in pooled liver samples from CCl_4_-treated KLF11^-/-^ mice and CCl_4_-treated wild type mice normalized to the their respective olive oil (OO) controls. Regardless of the genetic background, CCl_4_ treatment results in significant upregulation of collagen I. However, a number of TGFβ pathway markers are repressed only in the absence of KLF11, indicating that the fibrogenic response possesses KLF11-dependent and independent mechanisms. Levels of *ACTA2* are increased in the absence of KLF11, suggesting that the HSC response is mediated by this transcription factor. Numerical data may be found in Table S2.

HSCs represent only a small portion of liver tissue, the majority being comprised of endoderm-derived hepatocytes. To more precisely determine the effects of KLF11 depletion on mesenchymal cells, we utilized alpha smooth muscle actin (αSMA) labeling, a reliable and well-characterized marker of HSCs [18]. αSMA immunohistochemistry demonstrated an increase in αSMA-positive HSCs around the central vein and along the C-C and C-P septa (Figure 4A), indicating that HSC activation is linked to the observed increase in collagen deposition. Furthermore, quantification of the αSMA labeling showed that the HSC response is significantly increased in CCl_4_-treated KLF11^-/-^ compared to CCl_4_-treated wild type (p<0.05, Figure 4C). The percent of αSMA-positive HSCs in liver tissue observed for CCl_4_-treated wild type mice had a mean percentage of 4.26% when compared with that of 6.73% in CCl_4_-treated KLF11^-/-^ mice (p<0.05). Electronic microscopy also revealed disrupted cellular architecture in the CCl_4_-treated KLF11^-/-^ and wild type mice compared to OO-treated controls (Figure 4A). Analysis of αSMA, collagen 1A1, and collagen 1A2 expression levels by qPCR reflected that the level of transcript of each of these genes was also significantly increased in CCl_4_-treated KLF11^-/-^ livers compared to CCl_4_-treated wild type mice normalized housekeeping gene levels (Figure 5A). *ACTA2* levels were increased in in KLF11^-/-^ mice by 2.09 ± 0.10 fold compared to wild type. *COL1A1* and *COL1A2* levels were enhanced in KLF11^-/-^ mice by 16.16 ± 0.72 and 1.56 ± 0.10 fold, respectively, compared to wild type. Collectively, these experiments reveal an augmented fibrogenic response in the absence of KLF11 with an increase in HSC activation and corresponding activation of αSMA and collagen genes with collagen fibril deposition, C-C and C-P septa formation, and pseudo-lobule nodule formation. These data, together with the observations that KLF11 levels decrease during fibrosis, reveals that this transcription factor antagonizes fibrogenic responses in the liver. These data suggest that the role of KLF11 in the fibrogenic response is predominantly executed through deregulation of collagen secretion by mesenchymal cells.

### Expression of key genes for the global fibrotic response is modulated by KLF11

We next asked whether the global fibrotic response following chronic CCl_4_ treatment differs in the presence or absence of KLF11. Utilizing the same panel of 84 fibrosis markers as examined in Figure 3C, we compared the fibrotic response following chronic CCl_4_ treatment in KLF11^-/-^ and wild type mice (Figure 5B, Table S2). Under the conditions of chronic CCl_4_ treatment in both wild type and KLF11^-/-^ mice, 12 markers were significantly activated (36.4%) and 2 repressed (12.5%), representing the KLF11-independent, common fibrotic response to CCl_4_ treatment. However, 21 targets were activated (64%) and 14 repressed (87.5%) in either CCl_4_-treated wild type or KLF11^-/-^ mice, representing those targets that are directly or indirectly dependent on the presence of KLF11. As such, using a focused panel of critical fibrosis response markers, expression of key genes for the fibrotic response is modulated by the presence of a functional KLF11 transcription factor. Furthermore, the HSC activation marker αSMA was increased 8.1 fold in CCl_4_-treated KLF11^-/-^ but remained unchanged in CCl_4_-treated wild type animals (Table S2). Furthermore, several TGFβ signal transduction genes (Smad2, Tgfbr1, and Tgif1) have lower levels of transcript in CCl_4_-treated KLF11^-/-^ livers compared to the same treatment in wild type animals, confirming that KLF11 may work, at least in part, by modulating this pathway (Figure 5B). These results are congruent with the increased severity of fibrosis observed in Figure 4A in the absence of KLF11 following CCl_4_ treatment. Together, these data demonstrate for the first time that animals carrying a deletion in the KLF11 gene display an enhanced mesenchymal response to chronic injury, accompanied by an increased deposition of collagen.

### Additional alterations in the liver of KLF11^-/-^ mice are observed in response to chemically-induced injury

**Figure 6:**
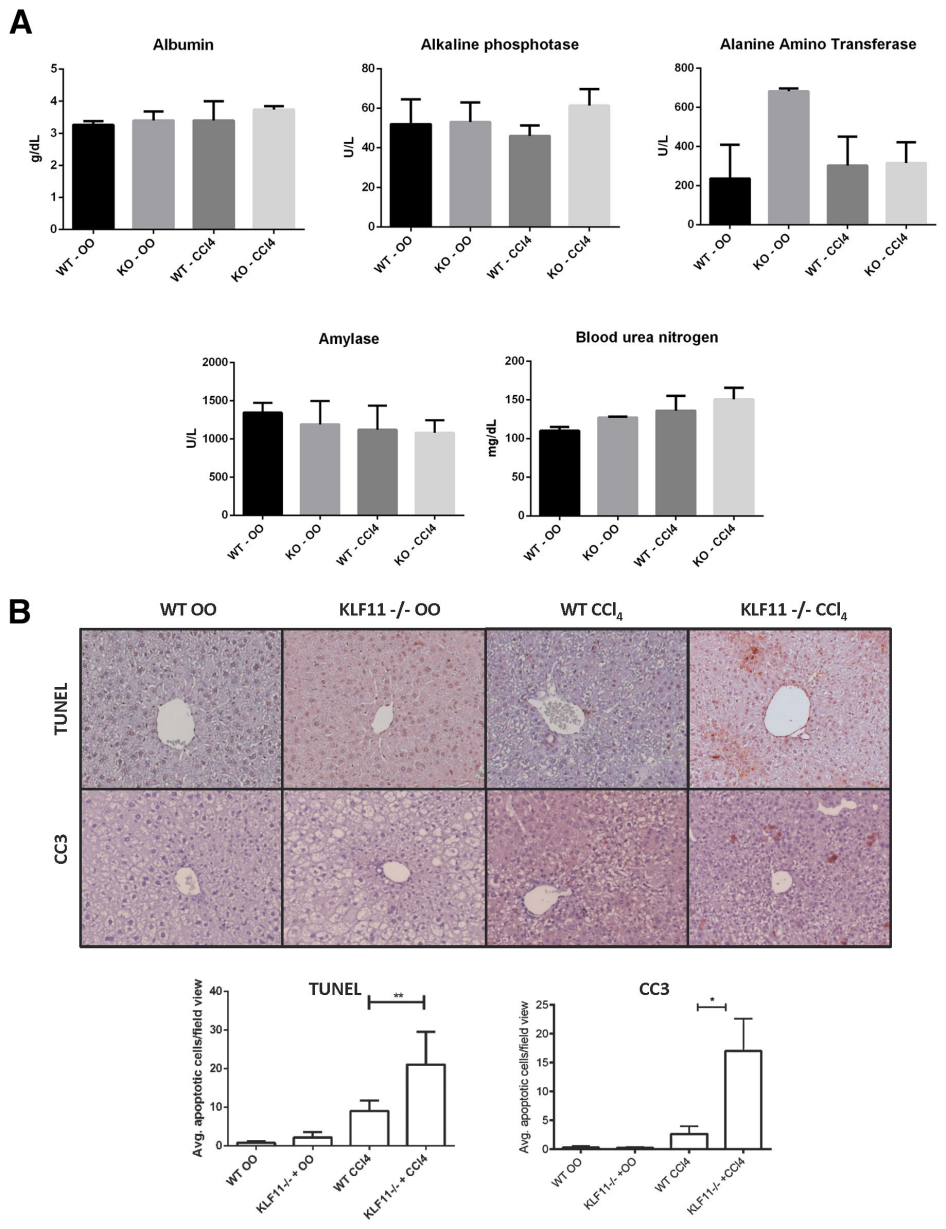
KLF11 inactivation does not affect liver function but increases hepatocyte apoptosis. (A) In a subset of olive oil and CCl_4_-treated KLF11^-/-^ and wild type mice, sera were screened for a panel of liver injury markers (as detailed in results). No differences were found between wild type and knockout animals under CCl_4_ treatment for the displayed markers. A significant difference was found between wild type and knockout animals under olive oil treatment owing to a previously characterized defect in fatty acid oxidation in the absence of KLF11. * = p<0.05. (B) Immunohistochemistry for cleaved caspase 3 and TUNEL quantification reveals that CCl_4_-treated KLF11^-/-^ experienced increased apoptosis, exclusive to hepatocytes, compared to CCl_4_-treated wild type animals. * = p<0.05. ** = p<0.01. All images appear at X200 magnification with scale bar equaling 200µm.

The potential exists for the KLF11-mediated portion of the fibrogenic response to be mediated through other cells types, including inflammatory macrophages. To test if the observed *in vivo* effects are secondary to differences in liver injury and/or inflammation in the presence of genetic permutation, we examined a panel of fifteen serological markers of liver function. No significant differences were found in 14/15 markers under the four conditions tested for albumin, alkaline phosphatase, amylase, bile acids, blood urea nitrogen, calcium, cholesterol, gamma globulin, creatinine, glucose, potassium, sodium, phosphate, or bilirubin concentrations (Figure 6A and data not shown). Alanine amino transferase demonstrated a significant difference between wild type and knockout conditions in the presence of olive oil alone. This result is concordant with previous observations about KLF11-mediated fatty acid oxidation [19]. From these data, we then conclude that no difference in the inflammatory response exists between wild type and KLF11^-/-^ mice, indicating that mesenchymal cell dysfunction in the absence of the transcription factor is one of the primary determinants of increased extracellular matrix deposition in response to injury.

Previous studies have demonstrated that fragmented DNA from hepatocytes undergoing apoptosis can stimulate stellate cell activity, including collagen deposition [20]. Two widely used apoptosis markers, namely TUNEL labeling and immunohistochemistry for cleaved caspase 3, revealed a significant increase in cell death in the hepatocytes of CCl_4_-treated KLF11^-/-^ livers compared to treated wild types (Figure 6B). As such, the possibility exists that pathobiological mesenchymal cell activation may occur directly through the de-repression of the collagen promoter in the absence of KLF11 or indirectly through the stimulation of collagen I synthesis by other transcription factors in response to hepatocyte apoptosis, a process heightened in the absence of KLF11 and therefore still KLF11-dependent.

In summary, in the current study, we have demonstrated that KLF11 functions as a critical regulator of mesenchymal cells in a variety of gene programs, but in particular, extracellular matrix remodeling. The function of KLF11 in this setting was defined using both cultured mesenchymal and a genetically engineered mouse model. We find that the manipulation of KLF11 levels impact on the expression of several ECM genes and define a role for this transcription factor as a silencer of collagen I expression. Further studies reveal that KLF11 executes this function primarily by coupling to the HP1-HMT chromatin-remodeling pathway. Depletion of KLF11 *in vivo* leads to mesenchymal cell dysfunction in the anti-fibrogenic response to liver injury. Thus, collectively, these studies assign a new function to KLF11 as a transcription factor that modulates mesenchymal cells function and participates in the pathobiological deregulation of these cells in an experimental model of liver fibrosis.

## Discussion

KLF11 is one of the best-characterized members of the family of TGFβ-inducible Krüppel-like factors that participates in the regulation of genes involved in cellular growth, inflammation, and differentiation [10,11,21,22]. The role of KLF11 in the function of epithelial and neuronal cells is well documented [11,13,20,23,24,25,26]. However, until the current study, a role for KLF11 in mediating the function of mesenchymal cells had remained unexplored. Here, we have utilized the liver stellate cell as a model mesenchymal system, where KLF11 drives the expression of multiple gene programs, including regulators of growth factor signaling, angiogenesis, apoptosis, and differentiation, among others. Predominant in this repertoire of biological functions is extracellular matrix remodeling. In particular, we show that overexpression of KLF11 in cultured stellate cells leads to a significant repression of collagen I expression and activation of matrix metalloproteinases. From this data, we hypothesized that if the physiological function of KLF11 in mesenchymal cells is to repress the expression of collagen I in response to stimuli, the *in vivo* levels of KLF11 will likely be altered during mesenchymal cell injury. Indeed, we observe that the exposure of mice to CCL_4_ results in a significant downregulation of KLF11 in the liver with concomitant de-repression of the collagen I promoter with increased expression and deposition of collagen fibrils. Note that the dynamic upregulation observed with collagen Ia1 in the CCL_4_ induced fibrotic liver (Figure 5A) is often regarded as a coordinated transcriptional response that accompanies collagen Ia2 regulation, which is likely the case in our experimental system. This data is congruent with a model whereby KLF11 is a critical brake point in the extracellular matrix remodeling function of mesenchymal cells in the liver, serving to tightly regulate the balance between synthesis and degradation. As fibrosis is a disease process in which the fine balance of synthesis and degradation is disrupted [27,28], we also hypothesized that mesenchymal cell function would be disordered in the absence of KLF11, leading to an unchecked deposition of collagen and increased fibrotic lesion formation. Indeed, using the same chemical model of liver injury and a genetically engineered KLF11 deficient mouse, we found that the absence of KLF11 leads to enhanced stellate cell activation with increases in extracellular matrix deposition. Furthermore, the severity of the fibrogenic response in KLF11 knockout animals is significantly more robust than that observed in wild type littermates under similar treatment. Thus, we conclude that one of the important new functions that can be assigned to KLF11 is to repress collagen fibril deposition in mesenchymal cells as a response to organ injury.

The biomedical importance of discovering a new KLF11-HP1-HMT pathway for the regulation of organ fibrosis lies in the fact that this process is one of the most common debilitating pathobiological events, which associates with significant morbidity and mortality by a myriad of diseases [29,30]. Moreover, a fibrogenic response (demoplasia) supports the growth of several cancers including pancreatic and hepatobiliary malignancies [31,32]. Thus, fibrosis-associated diseases kill millions of individuals in the U.S. and worldwide. Besides this costly human loss, fibrosis impacts our economy by reducing human productivity and increasing health care costs. Consequently, a better understanding of mechanisms that mitigate this process, such as the pathway described here, has significant biomedical relevance. Lastly, since fibrosis-associated organ dysfunction escalates with longevity, if not alleviated, this process will continue increasing as a severe health problem. In particular, our results contribute to extend the knowledge of the biology of stellate cells, which play a central role in the fibrosis of the liver and pancreas. Though stellate cells constitute only 5-8% of cells in these organs [33,34], upon fibrogenic stimuli, they proliferate and form fibrotic tissue that characterizes inflammation, cirrhosis, chronic cholangitis, pancreatitis, and hepato-pancreatic-biliary cancer-associated desmoplasia. Thus, the data provided here helps to increase our mechanistic understanding of human diseases, which are caused by stellate cell dysfunction.

The current study also contributes to extend the knowledge on molecular mechanisms that underlie mesenchymal cell dysfunction. For instance, our transcriptional analyses demonstrate that there are KLF11-dependent and independent portions of the fibrogenic response to chemical injury. Consequently, our experiments sought to shed light on how KLF11 mediates its effects using a combination of transcriptional assays and specific mutants. Our rationale for these investigations was based on the knowledge that gene expression is highly regulated by the cycle of chromatin dynamics initiated by the recruitment of transcription factors to specific sequences in promoters [35,36]. Upon binding, these proteins recruit chromatin enzymes which deposit posttranslational marks on histones to ultimately recruit chromatin effectors that determine either the activated (“on”) or silenced (“off”) state of a gene [37]. KLF11 binding to GC-rich sites can directly recruit the Sin3-HDAC pathway, which deacetylates local chromatin, dictating gene silencing [22]. However, KLF11 can also recruit HP1 and trigger HP1-HMT pathway, resulting in methylation of lysine 9 of histone H3, and triggering chromatin compaction to turn “off” the promoter [24]. Utilizing luciferase reporter assays, we found that expression of KLF11-EA29,30PP, which decouples KLF11 from the Sin3/HDAC pathway has no effect on the repression of collagen. However, expression of KLF11ΔHP1, which decouples KLF11 from HP1, results in complete loss of collagen repression, implicating the HP1-HMT pathway in executing the effects of KLF11 on collagen expression. Collectively, these data reveal that KLF11 likely mediates its antifibrogenic transcriptional program by directly binding to gene promoters along with specific cofactors, such as the HP1-HMT complex, a pathway which can be manipulated by small drugs currently in clinical trials.

In summary, the data in this report suggest that KLF11 is a critical transcription factor in the function of mesenchymal cells and, specifically, as a negative regulator of collagen secretion. The enhanced response to tissue injury in KLF11^-/-^ mice is likely initiated by failure of the KLF11-mediated brake on the fibrogenic response. This idea is further supported by our observation that, for CCl_4_-induced fibrosis to proceed in the mouse liver, KLF11 needs to be down-regulated. We provide mechanistic insights on how this protein works the cellular level, by describing a new function in mesenchymal cells. We also define molecular mechanisms for the function of KLF11 in the regulation of extracellular matrix by describing its ability to bind to collagen promoters and couple to the HP1-HMT pathway. Lastly, since small drugs which target this pathway are being tested in clinical trials for other diseases, the data reported here provide the first rational for the future design of new therapeutic interventions to ameliorate organ fibrosis, and thereby acquire significant biomedical relevance.

## Supporting Information

Table S1 **Transcriptional profiling of KLF11 overexpression in cultured liver mesenchymal cells.** Adenoviral-based overexpression of KLF11 in a cultured hepatic stellate cell lines using a 233 gene expression panel spanning growth factors, signaling pathways, adhesion molecules, extracellular matrix regulators, apoptosis molecules, and angiogenic mediators, among others. Fold changes are in relation to empty vector control.
(XLS)

Table S2 **Transcriptional profiling of KLF11 wild type and knockout mice exposed to CCL_4_.** Livers from olive oil and CCl_4_-exposed KLF11 wild type and KLF11^-/-^ mice were examined for transcriptional changes in a panel of common fibrosis markers. Fold changes are in relation to olive oil control for each condition examined.
(XLS)
